# Functional and Prognostic Assessment in Comatose Patients: A Study Using Somatosensory Evoked Potentials

**DOI:** 10.3389/fnhum.2022.904455

**Published:** 2022-07-04

**Authors:** Andrea Victoria Arciniegas-Villanueva, Eva María Fernández-Diaz, Emilio Gonzalez-Garcìa, Javier Sancho-Pelluz, David Mansilla-Lozano, Tomás Segura

**Affiliations:** ^1^Escuela de Doctorado, Universidad Católica de Valencia San Vicente Mártir, Valencia, Spain; ^2^Neurophysiology Service, Hospital de Manises, Valencia, Spain; ^3^Neurology Service, Hospital General Universitario de Albacete, Albacete, Spain; ^4^Neurobiología y Neurofisiología, Facultad de Medicina y Ciencias de la Salud, Universidad Católica de Valencia San Vicente Mártir, Valencia, Spain; ^5^Neurophysiology Service, Hospital Universitario Infantil Niño Jesús, Madrid, Spain

**Keywords:** prognosis, somatosensory evoked potentials (SSEP), N20, N70, coma

## Abstract

**Aim:**

The functional prognosis of patients after coma following either cardiac arrest (CA) or acute structural brain injury (ABI) is often uncertain. These patients are associated with high mortality and disability. N20 and N70 somatosensory evoked potentials (SSEP) are used to predict prognosis. We evaluated the utility of SSEP (N20–N70) as an early indicator of long-term prognosis in these patients.

**Methods:**

This was a retrospective cohort study of patients (*n* = 120) admitted to the intensive care unit (ICU) with a diagnosis of coma after CA (*n* = 60) or ABI (*n* = 60). An SSEP study was performed, including N20 and N70 at 24–72 h, after coma onset. Functional recovery was assessed 6–12 months later using the modified Glasgow scale (mGS). The study was approved by our local research ethics committee.

**Results:**

In the CA and ABI groups, the absence of N20 (36% of CA patients and 41% of ABI patients; specificity = 100%) or N70 (68% of CA patients and 78% of ABI patients) was a strong indicator of poor outcome. Conversely, the presence of N70 was an indicator of a good outcome (AC: specificity = 84.2%, sensitivity = 92.7%; ABI: specificity = 64.2% sensitivity = 91.3%).

**Conclusion:**

Somatosensory evoked potentials are useful early prognostic markers with high specificity (N20) and sensitivity (N70). Moreover, N70 has additional potential value for improving the prediction of good long-term functional outcomes.

**Clinical Trial Registration:**

[https://clinicaltrials.gov/], identifier [2018/01/001].

## Introduction

One of the main challenges in intensive care units (ICUs) is the assessment of the long-term neurological prognosis of patients with coma after cardiac arrest (CA) and other causes of coma after severe acute brain injury (ABI), which is uncertain in most cases (Taccone et al., 2014).

One of the main causes of death is hypoxic-ischemic encephalopathy after CA and remains one of the main causes of disability in these patients (Sandroni et al., 2018). Post-CA syndrome is defined by diffuse brain damage, myocardial dysfunction, and systemic ischemia–reperfusion response (Taccone et al., 2014). It is considered an emergency (González-García et al., 2007; Stevens and Sutter, 2013) because its most frequent causes are traumatic brain injury and cerebrovascular disease, for which rapid action is required to avoid spreading damage.

It is known that neurophysiological tests, such as somatosensory evoked potentials (SSEP N20) and electroencephalogram (EEG), are the most commonly used techniques in altered states of consciousness of the critically ill patient; in combination, they can be very useful in the diagnosis and prognosis of these patients (Ferré et al., 2009). There are guidelines describing their use to assess the probability of patient recovery, such as the European Resuscitation Council, The American Academy of Neurology (AAN) (Nolan et al., 2021), and recommendations for the French Society of Clinical Neurophysiology (André-Obadia et al., 2018). It is an important posterior column-medial lemniscal pathway (which is related to mechanoreception and Proprioception) to perform a multimodal assessment of these tests before making a drastic decision to discontinue life (Lachance et al., 2020; Scarpino et al., 2020).

Somatosensory evoked potentials reflect cortical function, as they indicate the integrity of the somatosensory pathway (Lachance et al., 2020) (Images provided in Supplementary Material). The absence of N20 potentials is known to have a high predictive value for poor neurological prognosis (Robinson et al., 2003; Oddo and Rossetti, 2011; Comanducci et al., 2020; Endisch et al., 2020); however, their presence and correlation with a good prognosis are limited (Amantini et al., 2008; Lachance et al., 2020; Scarpino et al., 2020).

Some authors describe the use of middle-latency (Madl et al., 2000; Zandbergen et al., 2006) N70 SSEP potentials as an indicator of cortical function and its relationship to cortico-cortical interactions and the ascending reticular activating system (Lachance et al., 2020). N70 is particularly vulnerable to hypoxemia (Madl et al., 1996; Zandbergen et al., 2006). Several authors consider its presence especially in hypoxic-ischemic encephalopathy as a good indicator of long-term functional recovery (Zanatta et al., 2012; Del Felice et al., 2017; Endisch et al., 2020). The SSEP study should be conducted at least 24 h after CA onset [earlier determinations can be interfered with by the cooling-off period (Pardal-Fernández et al., 2014)], which can increase the false positive rate (FPr).

Currently, the role of N70 potentials in patients with coma secondary to structural lesions has not been clearly established. The technical variability and the different criteria for its application in the clinic, as well as the limited evidence of its use, make it little known (Rossetti et al., 2017). It is essential to consider that the mechanism of injury by etiology is different from that of coma after CA. Several authors describe a high specificity of N20 to predict a poor prognosis (Comanducci et al., 2020; Cronberg et al., 2020).

Our main objective is to assess the usefulness of N70 SSEP in combination with N20 SSEP to predict long-term neurological prognosis in patients after coma, either CA or ABI.

## Methods

We conducted a retrospective analysis of prospective recruitment at a single academic hospital center. The General University Hospital of Albacete maintains a prospective registry of all tests performed on critically ill patients. We used this registry to identify patients treated for three consecutive years.

### Patients

**FIGURE 1:**
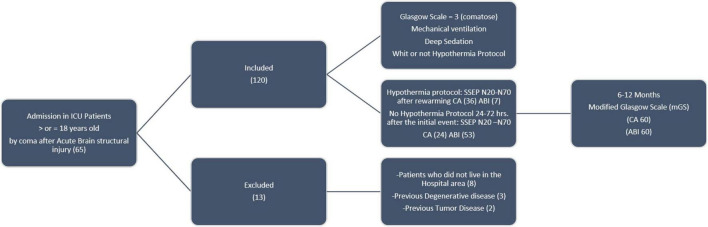
Methodology inclusion, exclusion of patients, and procedures.

Patients who were diagnosed with coma after CA or ABI and over 18 years of age were consecutively selected when admitted to the ICU of the General University Hospital of Albacete for three consecutive years. We excluded underage patients, those who did not live in the hospital area (to avoid problems in follow-up), and those who had a previous neurodegenerative disease (Figure 1).

### Procedures and Variables

All patients underwent a complete neurophysiological study as a routine practice. The study included N20 and N70 SSEPs in the first 72 h after CA or ABI (24–48 h or 48–72 h) and after rewarming if hypothermia was administered and not before 24 h after CA (Pfeifer et al., 2013). TH was applied according to a local protocol (temperature 33°Celsius for 24–48 h).

The SSEP study was conducted using XLTEK-Protector equipment according to a standard protocol with monopolar subdermal needle electrodes applied at the bilateral Erb point, the spinous process of C7 with anterior cervical reference, the bilateral shoulder and according to the international system 10–20 in Fz, C3, and C4 (Madl et al., 2000) with the reference in the ipsilateral auricular lobe. In all cases, stimulation was performed in the bilateral median nerve in the wrist with filters 30–3000 Hz (Erb-C5) 1–250 Hz (cortical recordings) analysis time of 100 ms (Guérit, 2005). Two block stimulations were performed. If the averages presented a significant artifact, muscle relaxant was intravenously administered. Peripheral (N9, N13, P14) and cortical responses (N20 and N70) were obtained. For this study, N20 and N70 SSEP results were dichotomized in the presence (when bilaterally present) or absence (absent on at least one side) of N20 and N70, respectively. Unilateral absence is an abnormal response (Rating 3, clinical interpretation of median nerve SSEP modified from Cruccu) (Koenig and Kaplan, 2015); therefore, the responses are dichotomized as bilateral present and unilateral or bilateral absent, and amplitude was not considered.

We assessed the effects of the following variables on the neurological outcome: gender, age, treatment of sedation: fentanyl, midazolam, or fentanyl and midazolam, treatment hypothermia: protocol for 24–48 h to a goal temperature of 32–33°C, time from coma onset to acquisition of the SSEP (between 24 and 72 h), duration of cardiorespiratory arrest before resuscitation (considered prolonged if longer than 20 min), place of CA (hospital or out-hospital) or type of brain injury (traumatic, subarachnoid hemorrhage, others: stroke, brain vascular malformations), in CA etiology cardiac, respiratory similar others studies (Chen et al., 2018) temperature, and blood test parameters (glucose > 200 mg, sodium, potassium, urea, and creatinine).

Finally, the patients’ long-term functional and neurological outcomes were evaluated between 6 and 12 months after the initial assessment. For this purpose, we used the modified Glasgow Scale (mGS) (Wilson et al., 1998) similar to other studies (Rothstein, 2000; Lew et al., 2003): 1 = death, 2 = persistent vegetative state, 3 = severe disability (conscious but dependent), 4 = moderate disability (disabled but independent), and 5 = no disability. An mGS score between 1 and 3 was considered to indicate an unfavorable outcome, and an mGS score between 4 and 5 was considered to indicate a good outcome.

### Data Collection

In our center, the patients’ medical information was included in a computerized hospital registry that was under the supervision of the Documentation Service. We reviewed these electronic medical records to obtain the information required for the study, and a follow-up was performed by telephone interview (independent evaluator). Data were analyzed in protected clinical data systems and deidentified for final storage.

### Ethical Aspects

The relatives of the studied patients gave written informed consent for the administration of the neurophysiological tests that were performed during the patients’ stay in the ICU on admission to the critical unit. The study was approved by our local research ethics committee. Confidentiality of the information was maintained throughout the study.

### Statistical Analysis

Patients were separated according to their etiology into two groups: coma after CA or coma after other ABI.

In the descriptive analysis, the mean and standard deviation (SD) are provided for normal quantitative variables, and the number of patients and percentages are shown for qualitative variables.

For the statistical analysis, many dichotomous variables were used, taking into account the variables of previous studies and guidelines (Sandroni et al., 2014). These include N20/N70 (presence vs. absence), hyperglycemia (glucose level > 200 mg/dl), sedative medication (yes: fentanyl, midazolam or fentanyl, and midazolam or not), functional outcome (unfavorable vs. good), duration of CA (prolonged > 20 min or not), place of CA (out-of-hospital vs. intrahospital), and therapeutic hypothermia (TH) protocol (yes vs. no). The chi-squared test and Fisher’s exact test were used as appropriate to evaluate the associations of these dichotomic variables with functional outcomes. To assess quantitative variables, Student’s *t*-test was used. For all comparisons, the level of significance was *p* < 0.05, and statistical precision was determined by the 95% confidence interval (CI). We used binary logistic regression in both groups in relation to neurological prognosis.

The diagnostic accuracy for predicting long-term functional outcomes using each test (N20, N70) was estimated using sensitivity (Se), specificity (Sp), positive predictive value (PPV), negative predictive value (PNV), and FPr; the values for each of these parameters are shown with their 95% CIs.

The data were analyzed using SPSS Software v.22 (SPSS Inc., Chicago, IL, United States).

## Results

A total of 120 consecutive patients were included in the study; there were 60 patients in each group (CA and ABI) and the number of patients was not limited. Coma after CA or ABI is a severe condition with an overall unfavorable outcome (74.1%) and high mortality (54.1%). Thirteen patients were excluded (Figure 1).

### Coma After Cardiac Arrest

In the CA group, there was a predominance of male gender (49/60) and a mean age of 55.07 years (range 18–87 years). In this group, poor functional recovery mGS < 3, was detected in 41 patients (68.3%), and 35 patients died (58.3%). Sixty percent were under hypothermia, fifteen patients (25%) suffered prolonged CA and 8 (13.3%) had out-of-hospital CA. The complete description of the clinical characteristics is given in Table 1.

**TABLE 1:** Characteristics of the study population in cardiac arrest (CA) (*n* = 60).

Variables	Full CA cohort (*n* = 60)	Unfavorable outcome (*n* = 41)	Good outcome (*n* = 19)	*P* value
**Gender, male, *n* (%)**	49 (81,7%)	34 (69.4%)	15 (30.6%)	0.730
**Age, years**	55,07 (56–65)	62 (53–64)	58 (49–65)	0.467
**Cardiac arrest**				
Out of hospital	8 (13.3%)	6 (14.6%)	2 (10.5%)	0.505
**Cardiac arrest > 20 min**.	15 (25%)	12 (29.3%)	3 (15.8%)	0.214
**Etiology**				
Cardiac	35 (58.3%)	21 (51.2%)	14 (73%)	
Respiratory	7 (11.7%)	5 (12.2%)	2 (10.5%)	
Others	11 (18.3%)	9 (22%)	2 (10.5%)	
Unknown	7 (11.7%)	6 (14.6%)	1 (5.3%)	
**Hypothermia, yes, *n* (%)**	36 (60%)	21 (51,2)	15 (78.9%)	0.51
**Medication**				
Fentanest	4 (6.7%)	4 (9.8%)	0	
Midazolam	8 (13.3%)	5 (12.2%)	3 (15.8)	
Fentanest + Midazolam	20 (33.3%)	10 (24.4%)	10 (52.6%)	
Other	12 (20%)	10 (24.4%)	2 (10.5%)	
No	16 (26.7%)	12 (29.3%)	4 (21.1%)	
**Glasgow at admission**		3 (3–8)	3 (3–8)	
**Hyperglycemia**				
>200 mg *n* (%)	28 (46,7%)	21 (51.2%)	7 (36.8%)	
**N20. *n* (%)**				0.001
Presence	38 (63.3%)	19 (46.3%)	19 (100%)	
Absence	22 (36.7%)	22 (53.7%)	0 (0.0)	
**N70. *n* (%)**				0.001
Presence	19 (31.7%)	3 (7.3%)	16 (84.2%)	
Absence	41 (68.3%)	38 (43.9%)	3 (15.7%)	
**N20 + N70. *n* (%)**				0.001
Both present	19 (31.7%)	3 (7.3%)	16 (84.2%)	
Both absent	22 (36.7%)	22 (53.7%)	0 (0.0)	
**Time of test**				
24–48 h	49 (81.7%)	33 (80.5%)	16 (84.2%)	0.516
>48 h	11 (18.3%)	8 (19.5%)	3 (15.8%)	

*Medication-other: antiepileptic, propofol, antibiotics.*

The diagnostic accuracy of N20 and N70 were analyzed both separately and together (Table 2).

**TABLE 2:** Diagnostic accuracy of somatosensory evoked potentials (SSEP) (N20 and N70) for long-term outcome prediction in patients with coma after cardiac arrest (CA) and after acute brain injury (ABI).

Test	Outcome	% Se (95% CI)	% Sp (95% CI)	% PPV (95% CI)	% PNV (95% CI)	% FP (95% CI)
**Cardiac arrest (CA)**						
**N20**						
Presence	Good	100 (83.2–100)	53.7 (38.7–67.9)	50 (34.8–65.2)	100 (85.1–100)	46.3 (32.1–61.3)
Absence	Unfavorable	53.7 (38.7–67.9)	100 (83.2–100)	100 (85.1–100)	50 (34.8–65.2)	**0.0** (0.0–16.8)
**N70**						
Presence	Good	84.2 (62.4–94.5)	92.7 (80.6–97.5)	84.2 (62.4–94.5)	92.7 (80.6–97.5)	7.3 (2.5–19.4)
Absence	Unfavorable	92.7 (80.6–97.5)	84.2 (62.4–94.5)	92.7 (80.6–97.5)	84.2 (62.4–4.5)	15.8 (5.5–37.6)
N20–N70						
Both present	Good	100 (83.2–100)	88.0 (70–95.8)	84.2 (62.4–94.5)	100 (85.1–100)	12.0 (4.2–30.0)
Both absent	Unfavorable	88.0 (70–95.8)	100 (83.2–100)	100 (83.2–100)	84.2 (62.4–94.5)	0.0 (0.0–19.4)
**Acute brain structural injury (ABI)**						
**N20**						
Presence	Good	100 (78.5–100)	54.3 (40.2–67.8)	40 (25.6–56.4)	100 (86.7–100)	45.7 (32.2–59.8)
Absence	Unfavorable	54.3 (40.2–67.8)	100 (78.5–100)	100 (86.7–100)	40 (25.6–56.4)	0.0 (0.0–21.5)
**N70**						
Presence	Good	64.3 (38.8–83.7)	91.3 (79.7–96.6)	69.2 (42.4–87.3)	89.4 (77.4–95.4)	8.7 (3.4–20.3)
Absence	Unfavorable	91.3 (79.7–96.6)	64.3 (38.8–83.7)	89.4 (77.4–95.4)	69.2 (42.4–87.3)	35.7 (16.3–61.2)
**N20–N70**						
Both present	Good	100 (70.1–100)	86.2 (69.4–94.5)	69.2 (42.4–87.3)	100 (86.7–100)	13.8 (5.5–30.6)
Both absent	Unfavorable	86.2 (69.4–94.5)	100 (70.1–100)	100 (86.7–100)	69.2 (42.4–87.3)	0.0 (0–29.9)

*se, sensitivity; Sp, specificity; PPV, predictive positive value; PNV, predictive negative value; FP, false positive rate. Statistically significant values are shown in bold.*

N20 was very useful in predicting unfavorable outcomes. We observed that all patients with absent N20 died or were in a persistent vegetative state between 6 and 12 months after CP, resulting in an Sp of 100% for predicting unfavorable recovery mGS < 3 with a FP of 0% but a low Se of 54% (PPV 100%, NPV 50%). Regarding N70, we observed that 84.2% of patients in whom N70 was present achieved good long-term outcomes mGS > 3, whereas 92.7% of those in whom N70 was absent had unfavorable recovery (Se 92.7%, Sp 84.2%, PPV 92%, PNV 84%). When both N20 and N70 were absent, the outcome was the same as when only N20 was absent (Sp and PPV 100%), but this analysis showed a significant improvement in predicting a positive outcome (Se 88% and NPV 84.2%). None of our patients had the absence of N20 with the presence of N70. Therefore, N70 determination was only useful when N20 was present. In this context, N70 improved the prediction of favorable recovery mGS > 3 considering that the presence of N70 only had an FPr of 7.3%, whereas the FPr of N20 was 46.3%.

The fifteen patients who recovered completely (mGS: 5) had a positive correlation with evoked potential results.

Binary logistic regression was evaluated (Table 3), fitting the model as predictors of good neurological prognosis: etiology of coma (to a lesser extent cardiac etiology), treatment with hypothermia, and presence of N70.

**TABLE 3:** Logistic regression-modified Glasgow coma scale and predictor of good outcome in patients with coma after cardiac arrest (CA) and coma after acute brain structural injury (ABI).

Group	Unadjusted OR (95% CI)	*P* value	Adjusted OR (95%)	*P* value
**Coma after cardiac arrest Predictor**				
Gender male. *N* (%)	0.00 (−)	0.711	−	
Age years	0.628 (−)	0.458	−	
Cardiac arrest Out of Hospital	181	0.663	−	
Cardiac arrest > 20 min.	0.025	0.262	−	
Etiology	0.00	0.084	0.001	0.997
Hypothermia treatment	117	0.41	182	0.998
Medication	48.82	0.667	−	
Glasgow at admission	0.00	0.524	−	
Hyperglycemia	750.6	0.787	−	
N20. *n* (%)	0.445 (−)	**<0.001**	−	
N70. *n* (%)	736	**<0.001**	651	0.993
Hypertermia	138.35	**0.041**		

**Coma after acute brain structural injury Predictor**	**Unadjusted OR (95% CI)**	***P* value**	**Adjusted OR (95%)**	***P* value**

Gender male. *N* (%)	1.15 (0.67−19.77)	0.542	−	−
Age years	0.957 (0.89−1.01)	0.080	−	
Cardiac arrest > 20 min.	1.691 (0.121−23,69)	**0.002**	−	−
Etiology	0.226 (0.053−0.958)	2.262	0.300 (0.089−1.014)	0.053
Hypothermia treatment	3.7 (0.049−293)	0.122	−	−
Glasgow at admission	1.451 (0.182−11.5)	0.326	−	−
N20	0.00 (0.00)	**<0.001**	0.00	0.998
N70	0.029 (0.001−0.675)	**<0.001**	0.042 (0.003−0.578)	**0.018**

*Statistically significant values are shown in bold.*

Finally, the absence of N20, the absence of N70, treatment without hypothermia, and hyperglycemia were associated with mortality (Table 1).

### Coma After Acute Structural Brain Injury

A total of 60 patients with post-ABI coma were included, of whom 48 (80%) were men, and the mean age was 55.07 years (range, 19–70 years). The causes of ABI were subarachnoid hemorrhage (*n* = 14, 23%), traumatic brain injury (*n* = 29, 48.3%), or other (stroke, subdural hematoma, or intraparenchymal hemorrhage) (*n* = 17, 28%). The severity of these patients’ conditions is reflected in the functional outcomes: 46 patients (76.7%) had a poor outcome mGS < 3, and only five patients (8.3%) were free of disability after 6–12 months. In this group of patients, the Therapeutic hypothermia protocol was not established. All data regarding these variables are shown in Table 4.

**TABLE 4:** Characteristics of the study population in acute brain structural injury (*n* = 60).

Variables	Full ABI cohort (n = 60)	Unfavorable outcome (n = 41)	Good outcome (n = 19)	*P* value
**Gender, male, N (%)**	48 (80)	36 (78.3)	12 (85.7)	0.426
**Age, years**	55,07 (56–65)	62 (53–64)	58 (49–65)	0.467
**Cardiac arrest**				
Out of hospital	8 (13.3%)	6 (14.6%)	2 (10.5%)	0.505
**Cardiac arrest > 20 min**.	26 (43.3%)	20 (43.5%)	6 (42.9%)	0.608
**Etiology**				
Trauma	29 (48.3%)	19 (41.3%)	10 (71.4%)	
SAH	14 (23.3%)	13 (28.3%)	1 (7.1%)	
Others	17 (28.3%)	14 (30.4%)	3 (21.4%)	
**Hypothermia treatment n (%) (yes**)	7 (11.7%)	5 (10.9%)	2 (14.3%)	0.522
**Glasgow at admission**		3 (3–5)	3 (3–8)	
**N20**. N (%)				
Presence	35 (58.3%)	21 (45.7%)	14 (100%)	0.001
Absence	25 (41.7%)	25 (54.3%)	0 (0.0)	
**N70**. N (%)				
Presence	13 (21.7%)	4 (8.7%)	9 (64.3%)	0.001
Absence	47 (78.3%)	42 (91.3%)	5 (35.7%)	
**N20 + N70.** N (%)				
Both present	13 (21.7%)	4 (8.7%)	9 (64.3%)	0.001
Both absent	25 (41.7%)	25 (54.3%)	0 (0.0)	
**Time of test**				
24–48 h	12 (19.3%)	8 (17.4%)	4 (21.4%)	0.516
>48 h	48 (80.7%)	37 (80.4%)	11 (78.6%)	

*Etiology-other: stroke, subdural hematoma, or intraparenchymal hemorrhage.*

The absence of N20 had the same associations in ABI as it showed in CA. Thus, high accuracy was found to predict poor functional outcomes mGS < 3, with 0% FP (Sp and PPV 100%) in our cohort. The presence of N70 was again associated with a good prediction of mGS > 3 outcomes (91.3% Sp), similar to the finding in CA (Table 2).

Binary logistic regression was evaluated (Table 3), with model fit for predictors of good neurological prognosis mGS > 3: etiology of coma (to a lesser extent trauma) and presence of N20 and N70 (*P* < 0.018).

### Somatosensory Evoked Potentials and the Modified Glasgow Coma Scale in Both Groups

Two cohorts of patients were assumed, each with 60 patients with different etiologies and mechanisms of coma. The use of SSEP in both groups was assessed. Regarding the results of the mGS in both groups, 87 patients (72.5%) had a poor prognosis, of which 39.2% had a bilateral absence of N20, and 66% had a bilateral absence of N70.

On the other hand, 33 patients (27.5%) had a good prognosis (p 0.414), 32 patients had the N70 present (26.7%).

## Discussion

This study supports that the absence of N20 was validated as an optimal marker of poor functional prognosis 6–12 months after admission in patients with postanoxic coma after CA or ABI; in these patients, N20 had a Sp of 100%. In both series, our results were similar to those of previous studies (Zandbergen et al., 2006; Daubin et al., 2008). However, the presence of the N20 response does not allow for the prediction of a good prognosis in these patients (Ferré et al., 2009).

On the other hand, the analysis of N70, especially when present, provides an added value in the good prognosis in contrast to N20. We have found some differences and similarities in reference to other authors who have already analyzed it.

Other studies have already analyzed the usefulness of N70, as is the case of the series published by Madl et al. (2000) in 162 patients with postanoxic coma. They described that the absence of this potential had an Sp of 97% and a Se of 94% to predict a poor functional prognosis. We found that N70 had a similar Se (92.7%) but a lower Sp (84.2%); we consider that this could be due to the differences in the evaluation times and cooling periods used in our study and in that of Madl et al. (2000).

The study of Zandbergen et al. (2006) concluded that N70 absence increased the accuracy of prediction of poor prognosis over results obtained using N20 by 18–48%. Our study did not show that N70 had any benefits over N20 for predicting negative outcomes, but our sample size was smaller (120 vs. 407 patients). Furthermore, the aforementioned study acknowledged some possible bias due to incomplete or insufficient data.

Other authors have analyzed N70, but the parameters for its collection and interpretation differ from ours. This is the case of Sherman et al. (2000), who consider N70 to be valid up to 176 ms (in our case, this value is absent), and the evaluation of SSEP was performed in the first 12 h, which, as previously mentioned, can lead to false positives and false negatives.

For patients in the ABI group, the Se of N20 was 54.3%, while that of N70 was 91.3% for an excellent functional outcome, indicating that the presence of N70 is more reliable than N20 (González-García et al., 2007). Interestingly, the Sp of N70 was much lower in ABI (64%) than in CA (84%). The data presented in our study are in agreement with those of previous reports (Madl et al., 1996), corroborating the existence of factors that may alter the results, as they could interfere with the somatosensory pathway and alter the acquisition of SSEPs (such as the transient absence of N20) in up to 15% of cases (Cruccu et al., 2008). However, this does not change the prognostic value of these tests, as most of these patients present with severe neurological sequelae (Guérit et al., 2009). Factors, such as edema, increased intracranial pressure, space-occupying lesions, and electrode placement in patients with extensive local damage (craniectomies or bone lesions), should be taken into account.

The absence of N20 (0% FP) predicts a poor prognosis in patients in both groups, and the presence of N70 increases the possibility of a favorable neurological evolution after CA (84.2% Se) and ABI (64.3% Se).

Among the study’s limitations, its retrospective design stands out. We were not able to evaluate other important prognostic factors, such as amplitudes; actually, decreased SSEP (N20) is associated with poor outcomes after CA (Barbella et al., 2020), neuroimaging (Scarpino et al., 2019), and neuron-specific enolase, or other tests, such as P300 or MMN. Another significant limitation of this type of study is the “self-fulfilling prophecy” bias. Although it is impossible to entirely exclude the possibility that the results obtained for SSEP could have led to the interruption of ICU care and therefore affected the odds of an unfavorable outcome, as far as we know, at the time this study was conducted in our hospital, the SSEP result alone carried little weight in decisions regarding withdrawal or maintenance of therapy. Another critical factor is TH, which can affect SSEP. We were careful to always obtain SSEP after the patient’s temperature was normalized.

The number of cases in the sample collection time, as well as the data analysis, could not be extended in time at the Albacete Hospital. We assume that both samples separately may not give conclusive results, as in other series (Zandbergen et al., 2006) where the study was multicenter.

## Conclusion

The main contribution of our research is that in patients with coma of neurological origin (either due to anoxia or structural damage) with the bilateral presence of N20, the determination of SSEP N70 is an early prognostic marker of good long-term functional outcome, which can increase its value when it complements other previously established prognostic markers, such as N20 and EEG. It is a simple and accessible technique that can be implemented within neurophysiological assessment in critically ill patients. The information we present can be of great value and can complement the multimodal decision when determining the long-term neurological prognosis of these patients.

## Data Availability Statement

The raw data supporting the conclusions of this article will be made available by the authors, without undue reservation.

## Ethics Statement

The studies involving human participants were reviewed and approved by Comité de Ética de la Investigación con Medicamentos (CEIm). The ethics committee waived the requirement of written informed consent for participation.

## Author Contributions

AA-V initiated and concluded the study. DM-L performed SSEP tests. TS supervised the design of the research and the neurological approach. EF-D contributed to the statistical design, reviewed the manuscript, and supervised its English translation. EG-G and JS-P oversaw the statistical study and its development. All authors read and approved the final manuscript.

## Conflict of Interest

The authors declare that the research was conducted in the absence of any commercial or financial relationships that could be construed as a potential conflict of interest.

## Publisher’s Note

All claims expressed in this article are solely those of the authors and do not necessarily represent those of their affiliated organizations, or those of the publisher, the editors and the reviewers. Any product that may be evaluated in this article, or claim that may be made by its manufacturer, is not guaranteed or endorsed by the publisher.
