# Supplementary figures and images for: Functional and Prognostic Assessment in Comatose Patients: A Study Using Somatosensory Evoked Potentials
Source: Front Hum Neurosci. 2022 Jul 4;16:904455. doi: 10.3389/fnhum.2022.904455 (PMC9289095; doi:10.3389/fnhum.2022.904455)

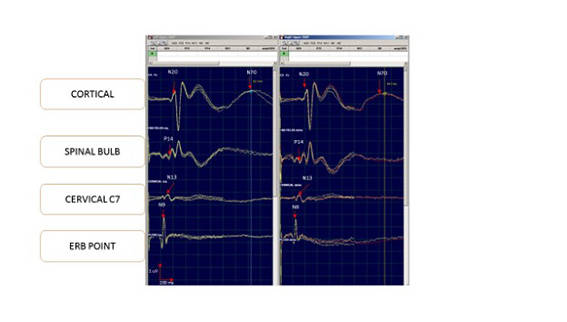

Supplement: Supplementary file 1 [file Image_1.JPEG]
